# Digital Integration and Automated Assessment of Eye-Tracking and Emotional Response Data Using the BioSensory App to Maximize Packaging Label Analysis

**DOI:** 10.3390/s21227641

**Published:** 2021-11-17

**Authors:** Sigfredo Fuentes, Claudia Gonzalez Viejo, Damir D. Torrico, Frank R. Dunshea

**Affiliations:** 1 Digital Agriculture Food and Wine Group, School of Agriculture and Food, Faculty of Veterinary and Agricultural Sciences, University of Melbourne, Parkville, VIC 3010, Australia; cgonzalez2@unimelb.edu.au (C.G.V.); fdunshea@unimelb.edu.au (F.R.D.); 2 Department of Wine, Food and Molecular Biosciences, Faculty of Agriculture and Life Sciences, Lincoln University, Lincoln 7647, Canterbury, New Zealand; Damir.Torrico@lincoln.ac.nz; 3 Faculty of Biological Sciences, University of Leeds, Leeds LS2 9JT, UK

**Keywords:** areas of interest, computer vision, sensory analysis, eye fixations, computer application

## Abstract

New and emerging non-invasive digital tools, such as eye-tracking, facial expression and physiological biometrics, have been implemented to extract more objective sensory responses by panelists from packaging and, specifically, labels. However, integrating these technologies from different company providers and software for data acquisition and analysis makes their practical application difficult for research and the industry. This study proposed a prototype integration between eye tracking and emotional biometrics using the BioSensory computer application for three sample labels: Stevia, Potato chips, and Spaghetti. Multivariate data analyses are presented, showing the integrative analysis approach of the proposed prototype system. Further studies can be conducted with this system and integrating other biometrics available, such as physiological response with heart rate, blood, pressure, and temperature changes analyzed while focusing on different label components or packaging features. By maximizing data extraction from various components of packaging and labels, smart predictive systems can also be implemented, such as machine learning to assess liking and other parameters of interest from the whole package and specific components.

## 1. Introduction

Packaging and labels are the first points of contact between food and beverage products with consumers. Around 95% of food and beverage products that do not have consumer preference assessments for packaging will probably fail in the market [1]. The implementation of new and emerging digital technologies for sensory analysis of food, beverage, and packaging products, such as video acquisition for physiological [2,3,4,5,6], emotional [7,8,9], and eye-tracking data [10,11,12], requires multiple devices from different companies and respective software packages for data acquisition, handling, and analysis [13]. The latter makes the data analysis process more complicated since it requires specialized personnel to simultaneously manage multiple devices and software, making the whole process time-consuming and cost-prohibitive. Hence, many studies focus on only one or a couple of biometrics at most, which are usually recorded independently [6,13].

The integration of several technologies is frequently not straightforward due to proprietary rights from different companies concerning their analysis algorithms or even images (e.g., FLIR for infrared thermal data). One computer application that has already integrated self-reported sensory data with infrared thermal imagery and visible video acquisition is the BioSensory App [14] developed by the Digital Agriculture, Food and Wine Sciences group (DAFW), The University of Melbourne (UoM), Australia. The BioSensory App can obtain, besides the self-reported data, digital information to extract (i) physiological biometrics from video of panelists, such as heart rate, blood pressure, and temperature changes; and (ii) emotional response from videos. The latter is capable of analyzing three head orientation parameters, eight emotions, valence, engagement, 21 different facial movements and 12 emojis that resemble the participants’ expressions.

Eye-tracking devices and software have been used as a tool to analyze the gaze of panelists when looking at imagery or video with multiple and varied applications, such as multimedia learning [15], aviation [16], tourism [17], and sports [18], among others. For food and beverages [19,20], eye tracking has been helpful in the research of warning labels on sugar levels [21], healthy labels and food choice [22], fixations in different areas of interest (AOI) [23], packaging design and type [24,25], and more complex situations, such as the influence of soundtracks on visual attention and food choice [26]. Other studies have combined eye tracking with contact sensors, such as electrodermal activity, to assess food perception [27]. However, contact sensors may introduce biases in the analysis due to participants’ self-awareness [13,28,29].

Combining eye-tracking and other remote sensing biometrics, such as emotional response, has been used primarily in psychiatric research, with some research interpreting only eye-tracking data with negative emotions [30]. In food and beverage labels, eye-tracking data have been combined with self-reported data such as wine purchase intention [31]. However, combining eye-tracking data with emotional responses based on video analysis using computer vision is rarer and mainly focuses on the overall assessment of the whole label [32].

This study aimed to propose the integration of eye-tracking information and emotional response of sensory panelists to assess specific areas of interest (AOI) of labels, such as images, logos, and nutrition information, among others, and self-reported liking of the overall label. The integration system proposed and trialed relies on the timestamp synchronization between the eye tracker device and the BioSensory App to create digital time tags for automated processing using multivariate data analysis.

## 2. Materials and Methods

### 2.1. Sensory Session Description

A total of 55 participants (44% males, 56% females; 25–50 years old) were recruited from the pool of staff and students from UoM. Power analysis was conducted using the SAS Power and Sample Size 14.1 software (SAS Institute, Cary, NC, USA), the result (1 − β > 0.999; effect size: 0.59) was used to confirm that the number of participants was enough to find significant differences between samples.

The sensory session was conducted in the Faculty of Veterinary and Agricultural Sciences laboratory from UoM and approved by the Human Ethics Advisory Group (Ethics ID: 1545786.2). The sensory laboratory, which was designed according to the ISO 8589 Sensory analysis—General guidance for the design of test rooms, has 20 individual booths with uniform lighting, and each is equipped with a Samsung Galaxy View 18” tablet (Samsung Group, Seoul, Korea) and a Gazepoint GP3 eye tracker (accuracy: 0.5–1.0 degree of visual, frequency: 60 Hz; Gazepoint, Vancouver, BC, Canada). The BioSensory application (App; The University of Melbourne, Parkville, Australia) [14] was used to display the questionnaire and to record videos of participants while evaluating the samples.

**Figure 1:**
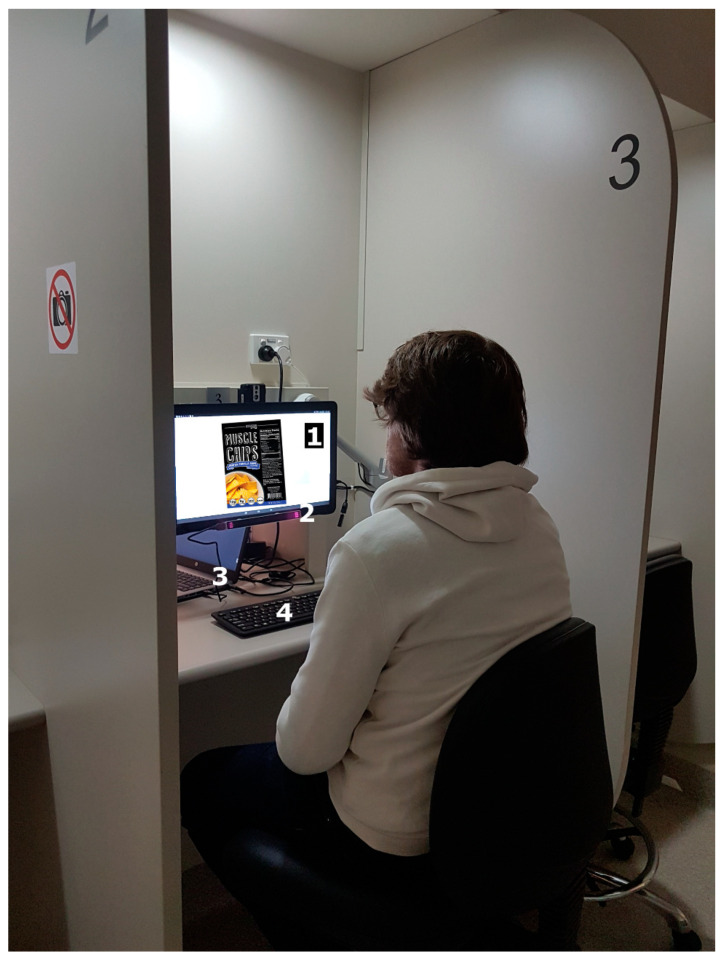
A participant during the sensory session in an individual booth equipped with (**1**) a Samsung 18” Tablet containing the BioSensory App, (**2**) a GazePoint GP3 eye tracker, (**3**) a computer connecting the eye tracker, and (**4**) a keyboard to switch between applications in the tablet. The FLIR infrared camera is also visible on top of the tablet but was not used in this study.

Three food labels (Stevia, Potato chips and Spaghetti) with different AOIs (product’s name, claims, nutrition facts, net content, nutrition squares, ingredients, image, manufacturer, suggested use, bar code, company logo and product’s denomination) were selected randomly and used as samples to test the new system proposed through the integration of eye-tracking and emotional response techniques. The eye tracker was connected to a computer, and the Gazepoint software presenting the slideshow with the samples was displayed in the tablet using RemotePC™ (RemotePC™, Calabasas, CA, USA). Participants were required to do a nine-point calibration between samples and were instructed to see the label for 10 s using the RemotePC App, while the BioSensory App was recording videos in the background. Once the 10 s looking at the label passed, a screen with instructions to switch to the BioSensory App was displayed. To do this, participants were provided with a wireless keyboard to switch between Apps (Figure 1). Once in the BioSensory App, participants had to rate the label for Overall liking (15 cm non-structured scale) and select the preferred AOI.

### 2.2. Biometrics

**Figure 2:**
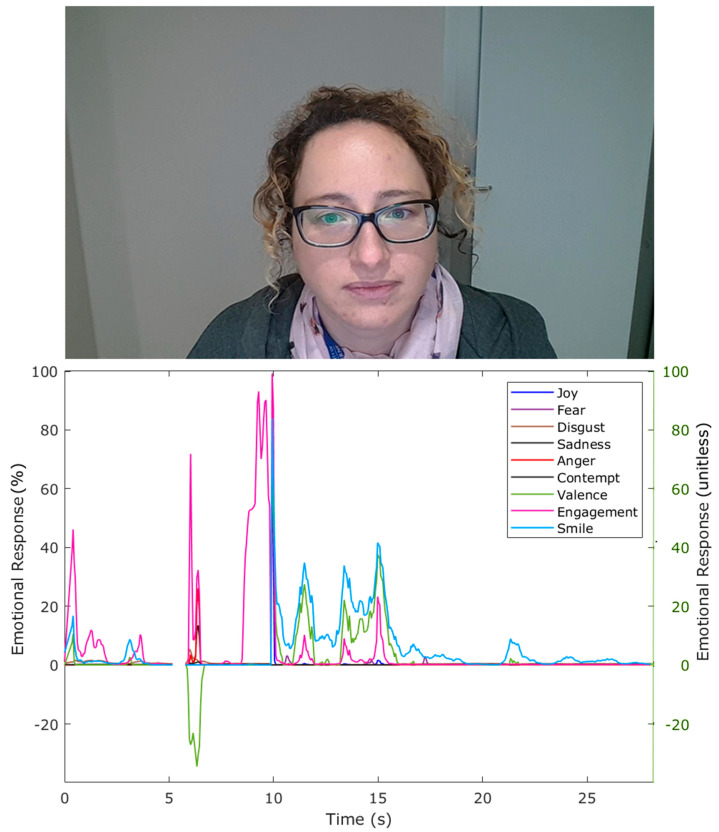
Example of a participant’s video and the emotion analysis plotted from the outputs of the software developed using Affectiva. Left (primary) *y*-axis corresponds to all emotions except for valence, while right *y*-axis (secondary) corresponds to valence (green).

Videos from participants were acquired using the BioSensory App and analyzed through a computer application developed by the DAFW from UoM based on the Affectiva software development kit (SDK; Affectiva, Boston, MA, USA; Figure 2). The parameters obtained from this analysis were emotions such as (i) joy, (i) fear, (iii) disgust, (iv) sadness, (v) anger, (vi) contempt, (vii) valence dimension, (viii) engagement, and (ix) smile facial expression.

Eye-tracking data was analyzed using the Gazepoint analysis software, and the parameters extracted per AOI for each participant were (i) time to first fixation, (ii) time viewed, (iii) fixations number, and (iv) revisits number.

Using the timestamps from both analyses, the emotional responses and eye-tracking data, the values of emotions were matched for each AOI to assess the participant’s reactions while viewing each area. Figure S1 in supplementary material shows an example of the emotions elicited per AOI.

### 2.3. Statistical Analysis

Data were analyzed for ANOVA to assess significant differences (*p* < 0.05) between samples using the Tukey honest significant difference (HSD) post hoc test (α = 0.05). Furthermore, a multivariate data analysis consisting of principal components analysis (PCA) and cluster analysis based on Euclidean distance was conducted using a customized code written in Matlab^®^ R2021a (Mathworks, Inc., Natick, MA, USA). A matrix was developed to assess significant (*p* < 0.05) correlations between emotional responses and the eye-tracking parameters using the latter software.

## 3. Results and Discussion

The analytical system proposed in this study allows the automated analysis of labels as a whole and to separate analysis from different label components. Below are presented the results from the new applications developed in the form of processed data for eye-tracking information and integrated analysis for eye tracking and emotional response based on videos from participants and computer vision algorithms.

The analyses presented in this paper are an example of how the data may be handled; however, each user of the proposed method would be free to analyze their own data according to their needs. ANOVAs may be conducted to assess differences per AOI as presented in this paper, but also per sample and the interaction of AOIs and samples; this will depend on the aim of the specific study.

### 3.1. Overall Label Liking End Emotional Response from Label Components

**Figure 3:**
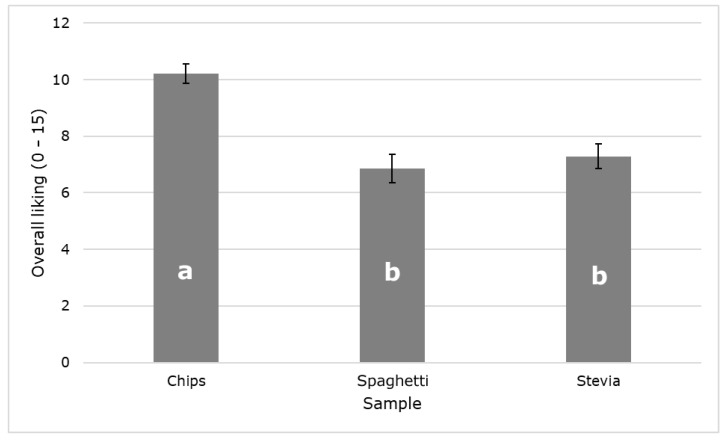
Mean values of the overall liking of the labels evaluated. Error bars represent the standard error. Different letters denote significant differences based on the ANOVA and Tukey honest significant difference (HSD) post hoc test (α = 0.05).

Figure 3 shows significant differences (*p* < 0.05) between samples for the overall liking. The chips label was the most liked, with the spaghetti and stevia labels being rated similarly. This may be due to the layout and colors of the labels and/or to the consumers preference for chips over spaghetti and stevia.

Table 1 shows the mean and standard error values of the emotional responses for each AOI. There were non-significant differences (*p* > 0.05) between AOIs for different emotions. However, the variability in standard error (SE) shows some trends that can be used to predict liking among other parameters using machine learning modelling [6,33,34].

### 3.2. Differences in Eye-Tracking Data for Label Components

**Figure 4:**
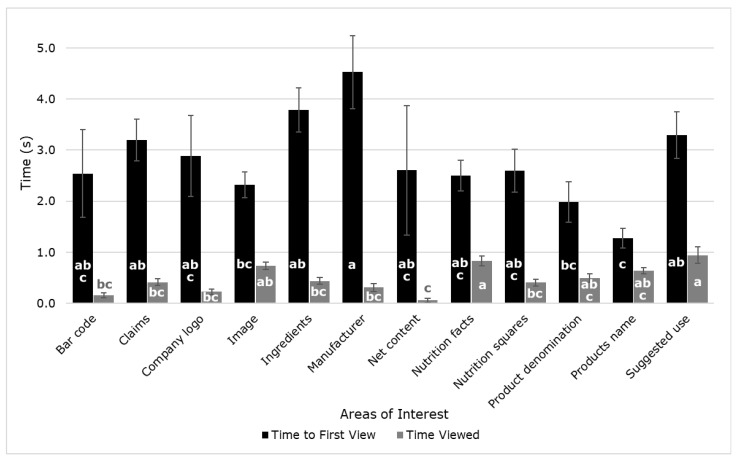
Mean values of the time to first view and time viewed from the eye-tracking analysis of the labels evaluated. Error bars represent the standard error. Different letters denote significant differences based on the ANOVA and Tukey honest significant difference (HSD) post hoc test (α = 0.05).

Figure 4 shows significant differences (*p* < 0.05) between samples for both the time to first view and time viewed. The AOI manufacturer was the one that took longer for participants to first view (4.53 s), which means it was the last AOI they see when evaluating the labels. On the contrary, the product’s name took the least time to be first viewed (1.28 s), this being the first AOI that participants focus visual attention on the labels analyzed. On the other hand, participants spent the longest time (0.94 s) viewing the suggested use than the other AOIs, with net content being the element they spent the least time (0.06 s). The large SE values were expected due to differences in participants reactions; this is since subconscious responses are being evaluated and stimuli elicit different responses in each individual.

**Figure 5:**
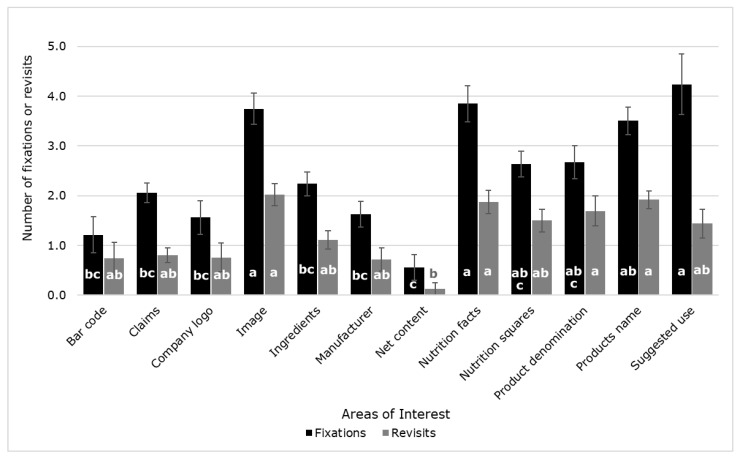
Mean values of the number of fixations and revisits from the eye-tracking analysis of the labels evaluated. Error bars represent the standard error. Different letters denote significant differences based on the ANOVA and Tukey honest significant difference (HSD) post hoc test (α = 0.05).

In Figure 5, it can be observed that there were significant differences (*p* < 0.05) between the AOIs for the number of fixations and revisits. Suggested use, nutrition facts, and image were the highest in the number of fixations (4.24, 3.85, and 3.75, respectively), while net content was the lowest (0.56). On the other hand, the image was the AOI with the most revisits (2.02), while net content had the least (0.13).

### 3.3. Integrating Eye Tracking and Emotional Response Data

**Figure 6:**
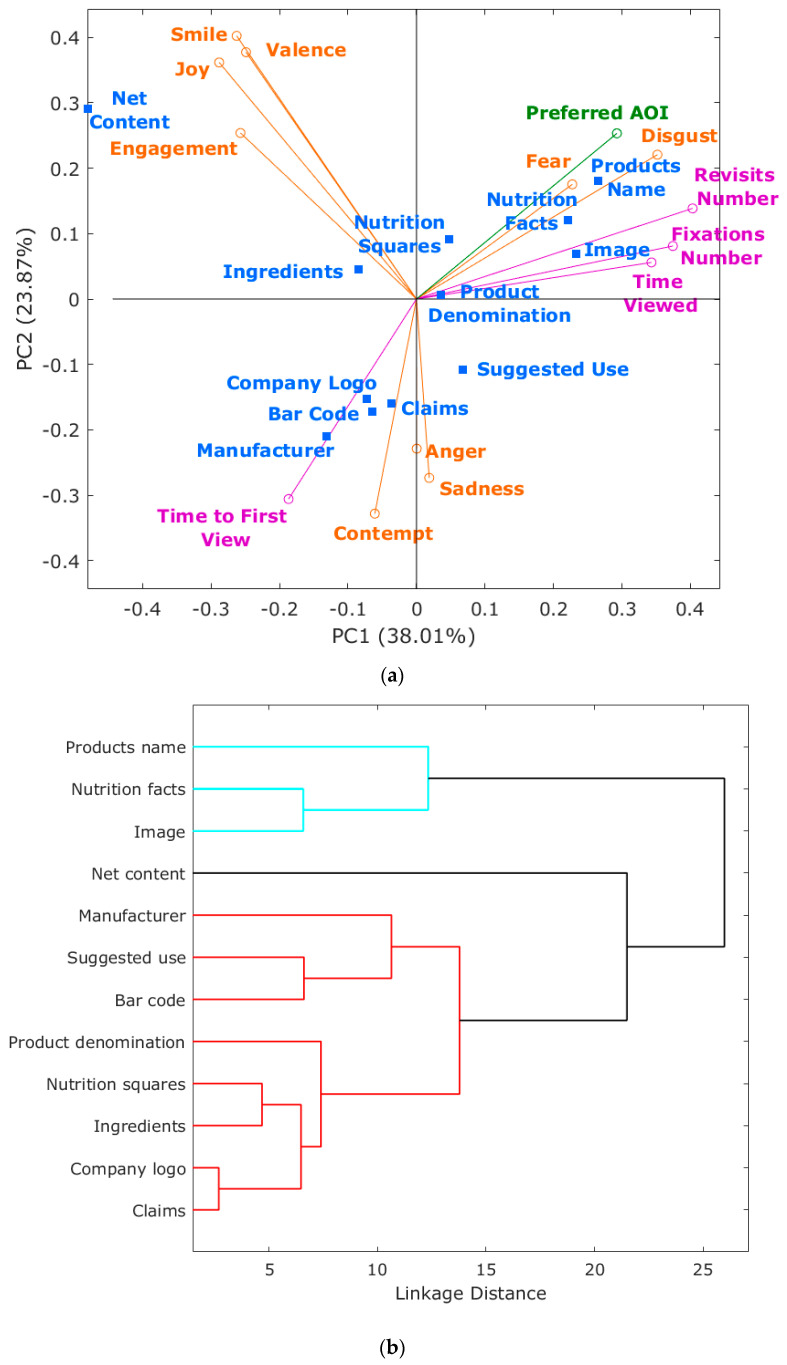
Multivariate data analysis based on (**a**) principal components analysis (PCA) and (**b**) cluster analysis. Abbreviations: PC: Principal Component; AOI: Area of Interest.

Figure 6 shows the combined data from eye trackers and emotional responses. Figure 6a shows that considering the first two principal components (PC), the PCA represented a total of 61.88% of data variability (PC1 = 38.01%; PC2 = 23.87%). According to the factor loadings (FL), PC1 was mainly represented on the positive side of the axis by the number of revisits (FL = 0.40), number of fixations (FL = 0.37), disgust (FL = 0.35), and time viewed (FL = 0.34). On the negative side, it was represented by joy (FL = −0.29), engagement and smile (FL = −0.26 for both). On the other hand, PC2 was characterized by smile (FL = 0.40), valence (FL = 0.38), and joy (FL = 0.36) on the positive side of the axis, and contempt (FL = −0.33), time to first view (FL = −0.31), and sadness (FL = −0.27) on the negative side.

The preferred AOI was positively related to fear, disgust, and number of revisits and negatively related to time to first view. Revisits number, fixations number, and time viewed had a positive relationship among them and disgust. Associated with these were the AOIs nutrition facts, image, and product name. This association coincides with results reported in an eye-tracking study to evaluate olive oil dressing labels, in which higher fixations were found for product’s name and image [25] and an eye-tracking study with organic food labels in which visual attention was higher when viewing the image [35]. On the other hand, time to first view was positively related to contempt, with AOIs manufacturer, bar code, company logo, and associated claims. Net content AOI was related to engagement, joy, smile, and valence. The other AOIs were more ambiguous as they are located more towards the center for the PCA. However, in Figure 6b, there are three main clusters, one of them with four subclusters. Product name, nutrition facts, and image conform one cluster; net content is independent of the other AOIs. The third cluster is composed of subgroups as (i) manufacturer, suggested use and bar code, (ii) product denomination, (iii) nutrition squares and ingredients, and (iv) company logo and claims.

**Figure 7:**
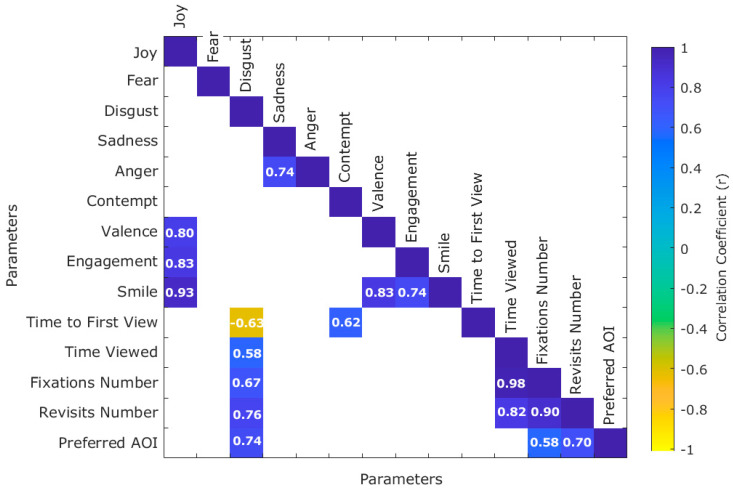
Matrix showing the significant correlations (*p* < 0.05) between emotional responses and eye-tracking parameters. Color bar represents the positive (blue) and negative (yellow) correlations.

Figure 7 shows there were positive significant correlations (*p* < 0.05) between disgust and time viewed (r = 0.58), fixations number (r = 0.67), revisits number (r = 0.76), and preferred AOI (r = 0.74). Similar results were found by Schienle et al. [36]; in their study, participants had a higher number of fixations when evaluating disgust images. Furthermore, disgust was negatively correlated with time to first view (r = −0.63). Whilst contempt was positively correlated with time to first view (r = 0.62). The preferred AOI had a positive correlation with fixations number (r = 0.58) and revisits number (r = 0.70). Engagement was positively correlated with smile (r = 0.74) and joy (r = 0.83) as expected. The latter was also correlated with valence (r = 0.80) and smile (r = 0.93). The correlation between valence, smile, and joy, also found in the PCA (Figure 6a), was expected as a positive valence is a measure of happiness [37].

### 3.4. Integration and Analysis of Eye-Tracking and Emotional Response

The BioSensory App used in this study was further developed through specific software modules for the post-analysis of videos acquired from panelists. One of those modules dealt with the integrated analysis of eye-tracking and emotional response output data by analyzing it based on timestamps and through a customized multivariate data analysis code for principal component (Figure 6a), cluster (Figure 6b), and correlation (Figure 7) analysis.

The use of multivariate data analysis such as PCA for the proposed system outputs to assess AOIs in labels may render critical information that may be picked up by the methods used separately. This may provide an overview of the specific AOIs from the labels that could require modifications in the design to satisfy consumers and, therefore, increase the overall acceptability of the labels. This is an advantage of the proposed system since the integrated method provides more precise information from consumers than traditional methods that use separate measures and focus on the overall emotional responses or other biometrics such as skin conductance elicited by the entire label [10,12,27]. This leads developers to fully redesign labels that may not be optimal to satisfy consumers and is more time-consuming and less cost-effective.

Not only self-reported data and emotional response can be integrated using the methodology proposed in this study, but also further digital data can be obtained with the BioSensory App system, such as physiological response based on heart rate, blood pressure, and temperature changes from panelists. The latter data were not presented in this study to avoid overcomplication of information presented. However, extra information can be used for more complex modelling strategies using artificial intelligence (AI).

The proposed system allows further analysis and the development of prediction models using machine learning techniques based on biometrics. The latter approach has been used in the case of consumer acceptability based on visual evaluation of beer pouring videos using eye-tracking, emotional and physiological responses [34] and for consumers acceptability towards beer tasting using biometrics such as emotions, heart rate, and body temperature [33]. Other authors have used machine learning modelling to predict food choice using eye-tracking gaze data when evaluating food images [38] and to predict participants age from their gaze patterns [39]. These digital and AI tools can be implemented in the design stage of packaging and labels rendering images or 3D representation of the same on screens for panelists or potential consumers. This could expedite the design and modification process since modifications can be readily assessed and applied digitally for immediate re-rendering. The latter will avoid the requirement of further sensory sessions and reduce costs. Previous research has shown that sensory analysis and liking of packaging and labels do not have statistical differences when packaging is rendered digitally on a screen compared to 3D physical prototypes for panelists to handle [40].

## 4. Conclusions

Further development of the BioSensory computer application has helped maximize the extraction of information from packaging and labels. The proposed system not only applies to the packaging and labels, but it can also give more specific information about the different components or areas of interest (AOI) and the overall acceptability of the products. A potential future application using artificial intelligence can be developed to assess which components are liked by consumers and which require modifications only from eye-tracking, facial expressions, and further biometrics. This AI system could expedite packaging design and secure the success of food and beverage products in the market.

## Figures and Tables

**Table 1:** Means (top value) and standard error (bottom value) of the emotional subconscious responses from consumers. Abbreviations: AOI: Areas of Interest.

AOI/Emotion	Bar Code	Claims	Company Logo	Image	Ingredients	Manufacturer	Net Content	Nutrition Facts	Nutrition Squares	Product Denomination	Products Name	Suggested Use
Joy	0.02	2.24	1.82	1.39	4.78	1.51	11.82	2.49	2.88	1.44	2.88	3.54
	±0.02	±2.24	±1.81	±0.83	±2.02	±1.51	±8.08	±1.12	±2.14	±0.88	±1.21	±2.44
Fear	1.82	0.53	0.42	1.18	2.83	0.01	0.07	1.35	2.15	1.52	3.04	1.10
	±1.82	±0.37	±0.41	±0.49	±1.16	±0.01	±0.07	±0.59	±1.26	±1.05	±0.91	±0.90
Disgust	0.47	0.65	0.56	0.99	0.44	0.44	0.31	1.21	0.92	0.52	1.61	0.60
	±0.10	±0.11	±0.10	±0.22	±0.04	±0.07	±0.04	±0.37	±0.43	±0.07	±0.78	±0.11
Sadness	0.09	0.80	0.91	0.08	0.11	0.02	0.02	0.15	0.03	0.43	0.17	0.65
	±0.07	±0.74	±0.86	±0.02	±0.05	±0.00	±0.01	±0.11	±0.01	±0.29	±0.11	±0.37
Anger	0.17	1.56	0.57	0.16	0.03	0.01	0.00	0.04	0.01	0.87	0.08	0.20
	±0.11	±1.52	±0.53	±0.11	±0.01	±0.00	±0.00	±0.03	±0.00	±0.83	±0.04	±0.12
Contempt	3.79	0.22	0.53	1.07	2.46	3.76	0.15	0.24	0.18	0.21	0.21	3.26
	±3.45	±0.03	±0.30	±0.66	±1.51	±3.10	±0.02	±0.06	±0.01	±0.02	±0.03	±2.00
Valence	−5.11	−0.94	−2.13	−0.52	2.11	2.03	11.67	0.79	1.46	2.70	0.51	−4.89
	±3.52	±1.95	±3.31	±1.00	±2.76	±1.99	±7.13	±1.68	±2.39	±2.06	±1.57	±3.03
Engagement	9.14	10.79	10.55	8.70	11.73	4.34	18.88	8.17	11.59	9.28	7.99	13.39
	±3.79	±3.34	±4.15	±1.87	±2.67	±2.94	±9.91	±1.89	±3.45	±2.85	±1.62	±3.67
Smile	3.06	2.25	2.44	3.03	7.19	2.82	12.58	4.80	4.01	5.39	4.59	4.15
	±1.72	±2.01	±1.82	±0.94	±2.22	±2.15	±7.40	±1.35	±2.26	±2.05	±1.34	±2.39

## Data Availability

Data and intellectual property belong to The University of Melbourne; any sharing needs to be evaluated and approved by the University.

## Supplementary Materials

The following are available online at https://www.mdpi.com/article/10.3390/s21227641/s1, Figure S1. Example of a heatmap from a label showing the different emotions elicited in consumers by each area of interest. In the top left, the identified eye section of participant is shown. The label has been blurred to hide brands and participant’s identity.
