# Supplementary material for: Digital Integration and Automated Assessment of Eye-Tracking and Emotional Response Data Using the BioSensory App to Maximize Packaging Label Analysis
Source: Sensors (Basel). 2021 Nov 17;21(22):7641. doi: 10.3390/s21227641 (PMC8622979; doi:10.3390/s21227641)
Supplement: Supplementary file 1 [file sensors-21-07641-s001.zip › sensors-1446854-supplementary.pdf]

sensors

MDPI

Article

Digital Integration and Automated Assessment of Eye-Tracking and Emotional Response Data Using the BioSensory App to Maximize Packaging Label Analysis

Sigfredo Fuentes [1,*], Claudia Gonzalez Viejo [1], Damir D. Torrico [2] and Frank R. Dunshea [1,3]

1 Digital Agriculture Food and Wine Group, School of Agriculture and Food, Faculty of Veterinary and Agricultural Sciences, University of Melbourne, Parkville, VIC 3010, Australia; cgonzalez2@unimelb.edu.au (C.G.V.); fdunshea@unimelb.edu.au (F.R.D.)
2 Department of Wine, Food and Molecular Biosciences, Faculty of Agriculture and Life Sciences, Lincoln University, Lincoln 7647, Canterbury, New Zealand; Damir.Torrico@lincoln.ac.nz
3 Faculty of Biological Sciences, University of Leeds, Leeds LS2 9JT, UK
* Correspondence: sfuentes@unimelb.edu.au

Citation: Fuentes, S.; Viejo, C.G.; Torrico, D.D.; Dunshea, F.R. Digital Integration and Automated Assessment of Eye-Tracking and Emotional Response Data Using the BioSensory App to Maximize Packaging Label Analysis. *Sensors* **2021**, *21*, 7641. https://doi.org/10.3390/s21227641

Academic Editor: Sara Tombelli

Received: 18 October 2021
Accepted: 15 November 2021
Published: 17 November 2021

Publisher's Note: MDPI stays neutral with regard to jurisdictional claims in published maps and institutional affiliations.

Copyright: © 2021 by the authors. Licensee MDPI, Basel, Switzerland. This article is an open access article distributed under the terms and conditions of the Creative Commons Attribution (CC BY) license (https://creativecommons.org/licenses/by/4.0/).

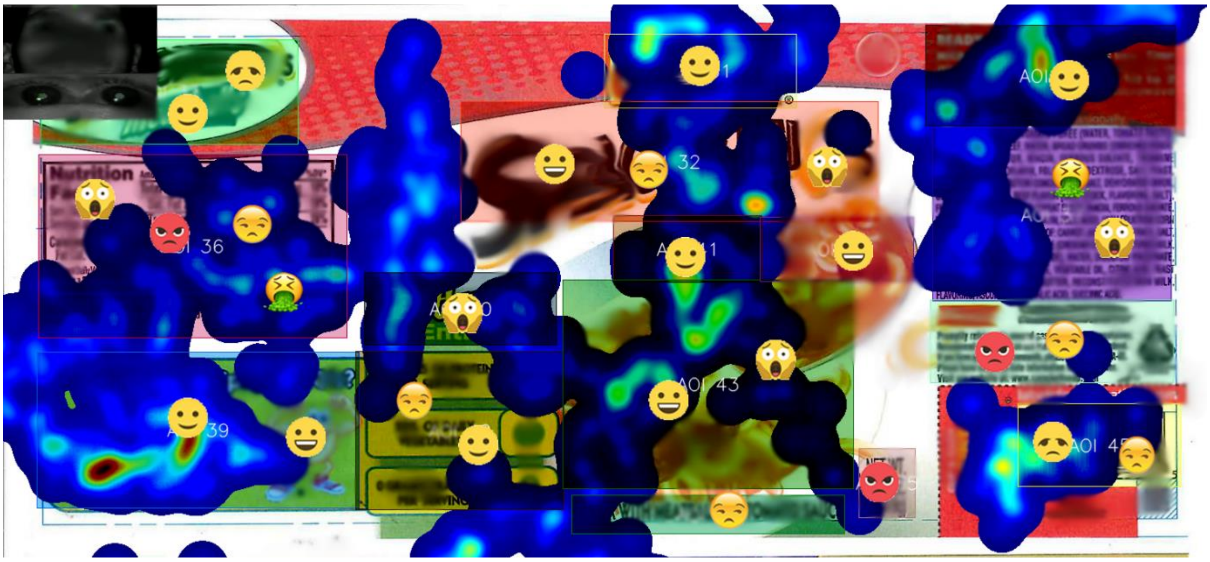

Figure S1. Example of a heatmap from a label showing the different emotions elicited in consumers by each area of interest. In the top left, the identified eye section of participant is shown. The label has been blurred to hide brands and participant's identity.
